# MOOCs as a massive learning resource for a Higher Education Community. The Universitat Politècnica de València experience using the EdX remote access program

**DOI:** 10.1007/s10639-022-11140-2

**Published:** 2022-06-20

**Authors:** Ignacio Despujol, Linda Castañeda, Carlos Turró

**Affiliations:** 1 Universitat Politècnica de València, Valencia, Spain; 2 Universidad de Murcia, Murcia, Spain

**Keywords:** MOOC, Higher Education, COVID-19, Case study

## Abstract

During the 2020 COVID-19 lockdown, edX launched an initiative, called the Remote Access Program, to give access to free certificates for its Massive Open Online Courses (MOOCs) to the communities of its partners. This paper describes the experience of a Higher Education (HE) institution in Europe participating in this initiative as a strategic action to spread MOOCs, not just as a resource to open the university educational offer but also to improve the professional development possibilities of its community. This case study explores general data about the participation of over 7,700 people (23,4% of the Universitat Politècnica de Valencia’s community) in these courses and data from participants (1,515) about their initiative perceptions. The data obtained evaluates how a university community integrates MOOCs into their day-to-day life under certain conditions and their perception of the quality and usefulness of the courses. Data shows most of the community did not have experience or knowledge about MOOCs (73%), they used it (they obtained 5,202 certificates, a 33% completion rate), they value the course quality (4.1/5) and are happy with the initiative (4.7/5), and they think MOOCs will be useful for their career (3.67/5) and will take more MOOCs in the future (98%, with 71% thinking it is worth paying for the certificate). These results remark the importance of thinking about MOOCs in HE Institutions not just as a punctual resource but as a strategic investment affecting the university teaching offer, the professional development of its community, and their social compromise.

## Introduction

Ten years after the first xMOOCs –the most instructivist version of the Massive Open Online Courses (MOOC)– were launched, many questions remain regarding the importance and opportunity of this educational phenomenon (Margaryan et al., 2015; Rolfe, 2015; Toven-Lindsey et al., 2015, among others). Some of these questions are related to the need for more critical visions on the interpretation of the role of MOOC in the Higher Education (HE) portrait (Bulfin et al., 2014) and others to the pedagogical implications of those “massive” teaching models (Bartolomé-Pina & Steffens, 2015).

Many universities worldwide started experimenting with MOOC courses years ago (Shah, 2013). Nevertheless, one of the big questions surrounding the universities’ commitment to developing MOOCs is to what extent these courses—their creation and use—can contribute to developing the aims of HE (Papadimitriou, 2020) and not only to its Uberification (Adell, Castañeda & Esteve-Mon, 2018).

When COVID-19 hit the world at the beginning of 2020, many countries enforced massive lockdowns to stop the spreading of the disease, most including school and university closures (Hale et al., 2021). In this scenario, MOOCs were an excellent option to address the need for quality online material for HE institutions (Ponce Ceballos and Ruelas Mexía, 2021; Duan, 2021).

The pandemic became a litmus test for MOOCs and their potential, not just as free resources for complementing formal education, but as strategical learning resources for professional development inside and outside institutions worldwide.

There were experiences of universities granting academic credit for MOOCs from other universities before the COVID-19 pandemic unleashed in 2021. Some integrate MOOC in blended courses (Khan, 2019), others accept micro master credentials as part of one of their masters (edX, 2017; MIT, 2021), some use them in interuniversity networks as a virtual exchange program (Delft University, 2021), and others let the students create a module of their bachelor studies using MOOCs (NUS, 2021).

When COVID-19 forced a lock-down in many countries, MOOCs were used to train teachers for the sudden transition to online learning (Boltz et al., 2021; Mays et al., 2021) or prepare sanitary personnel for COVID-19 (Quijano-Escate et al., 2020; Seale et al., 2021; Utunen et al., 2020).

Questions about what MOOCs bring to HE, once we have passed—albeit partially—that moment of “emergence”, are still there, and we need to take advantage of the experiences of these implementation moments to answer these questions or at least to coin them as experiences that will enrich our future work.

This paper describes the experience of Universitat Politècnica de València (UPV) using the Remote Access Program Initiative (RAP) from edX to spread the use of MOOCs as an alternative training possibility among its community members during COVID-19 lockdowns in 2020.

The data obtained evaluate how a university community integrates MOOC in their day-to-day life under the conditions of the COVID-19 lockdown, their participation, and their perception of the quality and usefulness of the courses. Some findings have exciting implications for educators and policymakers about how to integrate and use MOOCs as alternative learning resources.

## MOOC in HE, the fast trip until here

MOOC, the acronym for Massive Open Online Courses, was coined in 2008 by David Cormier and Bryan Alexander to refer to the course created by Stephen Downes and George Siemens, ‘Connectivism and Connective Knowledge/2008’ (CCK8), in which 2,200 students worked and learned actively (Siemens, 2012). It was popularised in late 2011 when Stanford University launched courses with over 100 thousand enrolments each (Rodriguez, 2012).

The considerable success of these first MOOC experiences brought media attention to MOOCs and was amplified, so 2012 was called, by some media, the year of the MOOC (Pappano, 2012). Some articles said MOOCs were the most important educational technology in 200 years (Regalado, 2012).

Nevertheless, the format used in this Standford’s courses, later called xMOOC, was different. It prioritised the use of videos explaining content and content review mechanisms in the form of self-correcting quizzes (Lugton, 2012) and some authors have characterised it as instructivist and individualist (Fidalgo-Blanco et al., 2016).

Soon, critics commented that MOOCs were nothing more than an incremental evolution of the educational technologies already available and that the dominant x-MOOC model introduced no pedagogical innovation, even stating the first MOOC was created in 1922 with open courses at the University of New York broadcasted using a radio station (Bartolomé-Pina, 2013). Some even considered that MOOCs were near the peak of inflated expectations of the Gartner Cycle (Schmidt, 2012).

Despite this, using MOOCs in HE followed a slow but steady path that accelerated in 2018. In those years, some of the most critical challenges for MOOCs were achieving self-sustainability and finding sources of revenue and ways to keep costs low (Conole, 2014; Schuwer et al., 2015; Shah, 2016). Some articles also pointed to four big barriers MOOCs had to overcome: developing revenue models to make the concept self-sustaining; delivering valuable signifiers of completion such as credentials, badges, or acceptance into accredited programs; increasing course completions; and creating ways to authenticate students, so it could satisfy accrediting institutions or hiring companies (Hill, 2012).

EdX started offering Micromasters in 2016 (micro-credentials that give access to academic credit), and Coursera followed in 2018 with Master tracks (McIntyre, 2018). During these years, the prominent MOOC platforms had been drifting from the “open” model, putting part of their content after a paywall (first the certificates, later the graded assignments) (Shah, 2017). The MOOC model in the big platforms has shifted towards a well-established business model of online postgraduate and continuing education, and most offer academic credit-bearing micro-credentials (Pickard et al., 2018).

With this level of success and development, it is hardly surprising the term MOOC has been the object of study in an impressively high number of literature reviews ranging from 2013 (e.g., Liyanagunawardena et al., 2013) until more recent times (e.g., Babori, 2020). Most of those works coincide to a point that seems the original problems have not been solved, and MOOCs are still struggling with low completion rates and the fact that most of its learners are educated individuals from the world’s most affluent countries rather than initial learners (Reich & Ruipérez-Valiente, 2019), making less clear than ever the real role of MOOCs in HE and if the effort is worth it.

Nevertheless, the MOOC movement has continued its almost exponential expansion, and at the end of 2020, there were over 16,300 courses from 950 universities worldwide with over 180 million enrolments (Shah, 2020), with thousands of platforms offering MOOCs (Open edX, 2018), ranging from country platform to small niche ones (Open edX, 2021).

## Case study context: The UPV and the edX remote access programme

UPV is a mid-sized public university that has committed over the last decade to use MOOC as one of its strategic levers to digital transformation, not only as digital resources to complement its educational offer but also as part of its social engagement (Despujol, Castañeda & Turro, 2018).

UPV joined edX –one of the leading MOOC worldwide platforms with over 160 partners, 3000 courses and 35 million users– at the end of 2014, and now this institution is the leader in its Spanish speaking offer, with over 100 courses and 3.5 million enrolments when writing this paper (edX, 2021).

In March 2020, when massive lockdowns started to be enforced worldwide to contain the spread of the COVID-19 pandemic, edX created a partner community where members could access the courses and programs of any other member at no cost. This initiative was called the “Remote Access Program” (RAP) (Randall, 2020a).

In a few days, over 60 edX partners from all over the world joined RAP (Randall, 2020b). UPV joined the programme understanding it as an opportunity to help other edX partners communities with the UPV generated MOOCs and as an excellent opportunity to offer external high-quality educational content to the UPV local community.

The free access was achieved using promotional codes that edX sent in CSV format text files to each customer institution by mail. All RAP participating members could ask edX for an unlimited number of codes during the initiative –through June 30 of 2020–as edX sent more codes when the institutions asked for them. These codes had to be distributed to the users of the customer institution. They could be redeemed when purchasing the certificates of the courses included in each initiative to get them for free.

UPV distributed 24,613 codes to 7,712 of its students, faculty and workers (own sources, from the statistics of the app used to distribute the codes), so around 23.4% of its 33,042 community members (UPV had 29,009 students, 2,579 faculty and 1,454 staff (U.P.V., 2021a, b)) asked for at least one code.

## Study

This study explores an innovative experience of using MOOCs as the primary learning resources (RAP) for a whole university community in the context of a HE Institution (UPV) during the COVID-19 crisis lockdowns. The study’s primary goal is to evaluate the experience and its potential consequences on the use of MOOCs in HE in a massive way.

For doing it, this study formulates the following research questions:

- RQ 1: Who participated in the experience?
- RQ2: What kind of courses do participants choose and from what institutions?
- RQ3: Does the experience increase the knowledge about the potential of MOOCs among the university community?
- RQ4: Is the use of MOOCs in a massive open way a satisfactory experience for participants?
- RQ5: Under what conditions would participants use MOOCs and certifications in the future?

The exploration has been structured as a case study, as it is a research approach to generate an in-depth, multi-faceted understanding of a complex issue in its real-life context (Crowe et al., 2011). This case study is considered evaluative but with a descriptive approach (Cohen et al., 2017).This means it explains and judges how the implementation of the MOOCs happened –evaluating it–, but providing a narrative account of the process –with a descriptive approach–, rather than a comparative one.

This study includes the narrative from the team that developed the institutional implementation of the experience, as described in the previous section of the study content, using three data sources: a survey to obtain a detailed description of participants' perceptions about their experience, the database of code requests, with the emails of the users, and the summary of the learning analytics of the initiative, provided by edX, to get the total number of codes redeemed and the total number of certificates obtained.

The survey included the collection of:

- Demographic information (age, gender, residence place, current job status and level of studies)
- Information about the initiative
  - satisfaction level, with a scale from 0 to 5 where 0 is the minimum
  - number of requested codes
  - number of used codes
  - number of obtained certificates
- Information about the motivations to participate
- About MOOCs
  - Previous knowledge about MOOC
  - Intention to take MOOC in the future
  - Reasons to choose a MOOC
  - Willingness to pay for MOOC certificates
  - Reasons to pay for MOOC certificates
- Some information about each one of the MOOCs taken (institution, name of MOOC, quality of the MOOC from 0 to 5, contribution to the professional career from 0 to 5, finalisation of the MOOC)

The structure and content of the survey were validated using a content and face validation (Holden, 2010) process that guarantee the appropriateness and relevance of the items as they appear to the persons answering the survey (Connell et al., 2018).

### Procedure

1,515 people from the 7,712 who asked for at least a code answered the survey (19.4%). The survey was sent at the end of September 2020, almost three months after the RAP initiative ended. Even if it is not a randomised sample, the percentage of the population and the range of ages and profiles included (data will be shown in the results part) makes them incredibly valuable for understanding the experience (Hibberts et al., 2012).

The data provided by edX shows that 15,744 of the codes provided to UPV were redeemed and that 5,202 certificates from different institutions were obtained (a 33% completion rate). It also shows that around 27% of UPV codes were used to get certificates for UPV courses.

Quantitative information has been analysed, calculating the averages and standard deviation of every answer of the survey. It has been studied differentiating university students and workers (including part-time students, alumni and university staff).

Also, the statistical inference values have been analysed (as p-values or size effect), testing if the hypothesis that the average of students’ and workers’ distributions are the same can be rejected, and it has been confirmed that p-values are smaller than 0.001 in the cases studied. Therefore, it theoretically confirms there is a difference between the average of both distributions (workers and students). Nevertheless, this study is not reporting the p-values obtained, considering the sample is not a random sample, so the “inference becomes tricky or outright impossible” (Hirschauer et al., 2021: 1), and researchers have preferred to maintain the importance of the size of the sample, rather than artificially randomising it.

Regarding comments on the survey, they were analysed under an analysis of content technique that classified and coded them as positive, neutral, or negative (Saldaña, 2015).

## Results

In this section, data and results are shown. Data have been organised using the Research Questions order.

### RQ 1: Who participated in the experience?

There were two main collectives involved in the initiative, University students and employees. But, even as the initiative was communicated only to the active members of the community, any email account from the upv.es domain was able to ask for codes of the initiative; therefore, former students, members of the alumni association –with an email from the alumni.upv.es domain–, could also participate. So, according to the email domain that requested codes, participation in the initiative among the community members groups was 77.4% students, 18.4% employees, and 4.2% alumni.

Regarding data collected with the survey, 60% of the responders were male and 40% female, and the average age was 29.4 years, with a peak in 22 years and another smaller and flatter peak in 50 years.

**Fig. 1:**
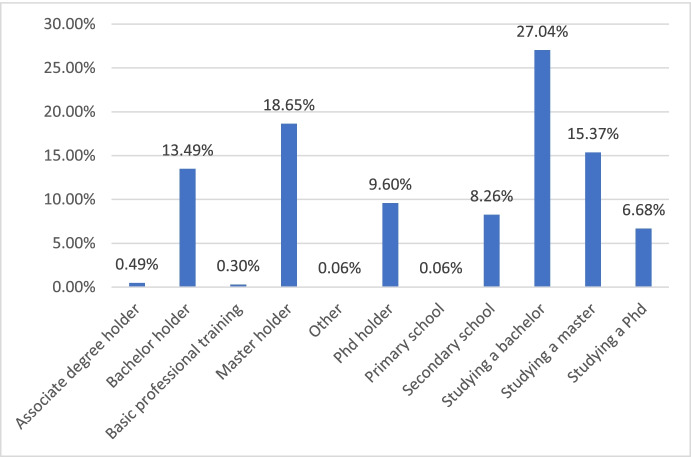
Level of education achieved

**Fig. 2:**
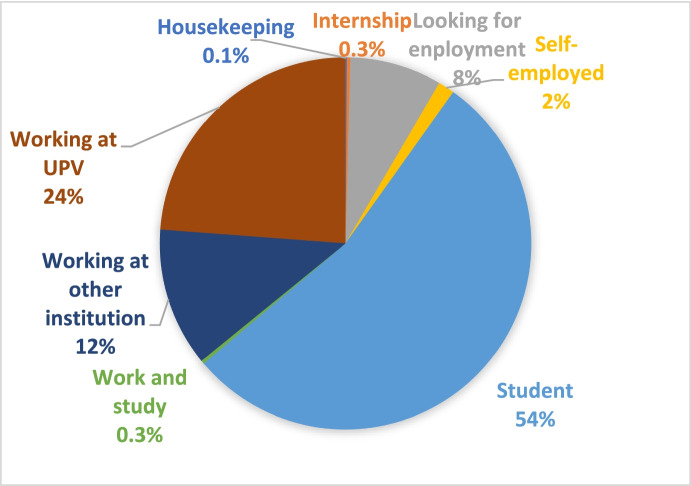
Employment status

Concerning the maximum level of education achieved by the respondents to the survey, we find two big groups, the ones that in the moment of the initiative were studying a bachelor, a master or a PhD (48.19%) and the ones that already had it (41.5%). Moreover, as is shown in Fig. 1, some people with primary or secondary education (10.31%) as their maximum level of education also participated in the initiative:

Concerning the employment status of participants, the answers in the survey show that 54.3% of participants define themselves as students or interns, 24% as working at UPV, 12.1% as working for other institutions, 8.45% as looking for a job, 1.6% as self-employed, 0.25% as working and studying and 0.07% as housekeeping as seen in Fig. 2.

Considering the anonymity of the survey –we do not have access to the respondents’ email–, the analysis of those answers regarding employment status using the level of education answers can give a more global perspective about the profile of respondents. Therefore, since 22.4% of respondents have answered they are working for other institutions, looking for a job, self-employed and/or housekeeping, using data from their level of education, we see that some are studying a bachelor, a master or a PhD, what identifies them as students that are working and studying part-time, and the rest are alumni. Therefore, considering the other answers regarding the employment status, 7.2% of this 22.4% can be considered students and 14.2% alumni. So, 61.75% of the responders were students (54.3 + 7.2 + 0.25), 24% UPV employees and 14.2% alumni.

The answers from these groups will be included in the same group as the working for UPV group when looking for differences between working people and students’ responses, given their working experience.

Most users (87.4%) resided in Comunitat Valenciana (the Spanish region where UPV is located), up to 95.2% including other regions of Spain, and the rest in different countries of Latin America and Europe (Ecuador 1%, Colombia 0.65%, México 0.42%, Germany 0.42%, Denmark 0.3%, UK 0.24%, Perú 0.24% or France 0.18%, for example).

### RQ2: What kind of courses do participants choose and from what institutions?

The answers to the survey include 3,237 ratings of 690 courses from 67 edX member institutions.

**Fig. 3:**
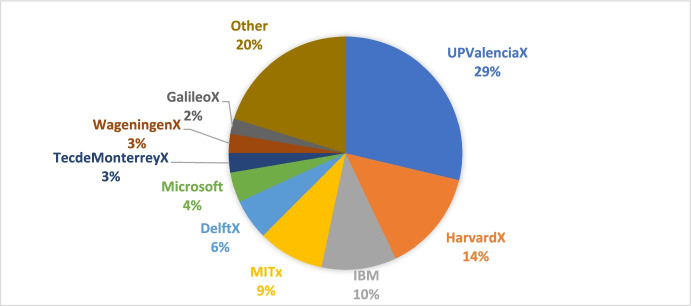
Percentage of courses per institution

The data provided by edX indicates 27% of the codes granted to UPV were used to get certificates from UPV courses. This figure is similar to the 28.7% obtained from the survey data and shown in Figs. 3.

As presented in Table 1, the most popular courses reported by users in the survey focus primarily on Excel (5), Python (7) and R(2), applied to data analysis, and the other 6 most popular courses cover Office 365 (1), engineering simulations (1), introduction to computer science (1), project management (1), Android programming (1) and stock investment (1), indicating that software tools for data analysis was the topic that most interested the participants.

**Table 1:** Number of enrolments per course in the 20 most popular courses

Course	Number of enrolments
Excel: gestión de datos (Excel: Data management)	115
Excel avanzado: importación y análisis de datos (Advanced Excel)	83
Excel: Fundamentos y herramientas (Excel: Fundamentals and tools)	71
Analysing Data with Python	52
Python: aprender a programar (Python: learn how to code)	43
Introducción al Office 365 (Intro to Office 365)	40
Statistics and R	39
Analysing and Visualising Data with Excel	39
Data Science: R Basics	38
Introducción a la inversión bursátil (Intro to stock invesment)	37
Python Basics for Data Science	37
CS50’s Introduction to Artificial Intelligence with Python	36
A Hands-on Introduction to Engineering Simulations	33
CS50’s Introduction to Computer Science	33
Machine Learning with Python: A Practical Introduction	32
Introducción a la gestión de proyectos (Intro to project management)	31
Introduction to Data Analysis using Excel	30
Using Python for Research	27
Visualising Data with Python	27
Android: Introducción a la Programación (Android: Intro to coding)	27

### RQ3: Does the experience increase the knowledge about the potential of MOOCs among the university community?

**Fig. 4:**
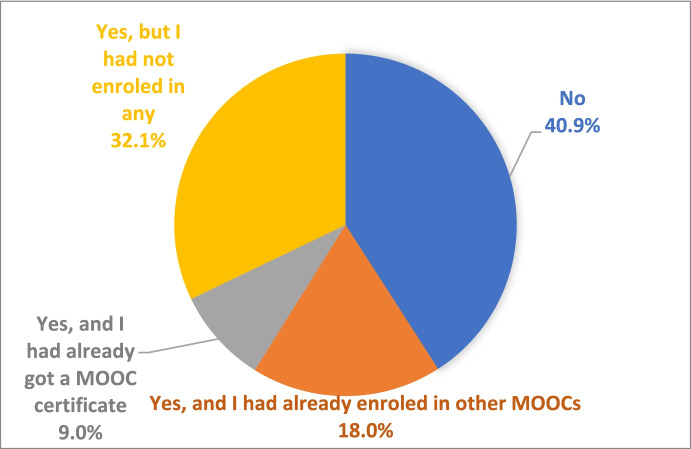
Previous Knowledge about MOOC

Regarding the survey answers, almost 41% of the respondents knew nothing about MOOCs before they participated in the initiative, and 32% knew about them but had not taken any, for a total, 73% of the users, as is evident in Fig. 4.

**Fig. 5:**
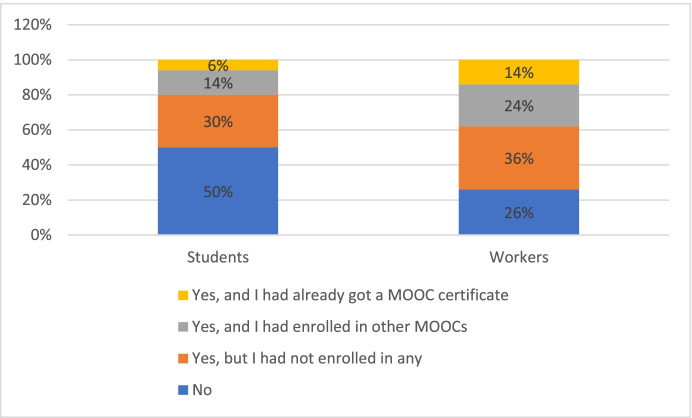
Previous Knowledge about MOOC by group

Differentiating students and workers' data, as shown in Fig. 5, 49.7% of students did not know about MOOCs before they participated in the initiative, and 30.2% knew about them but had not enrolled in any (a total of 79.9%). In the case of workers, 26% did not know about MOOCs in advance, and 35.4% knew about them but had not enrolled in one (a total of 51.4%).

### RQ4: Is the use of MOOCs in a massive open way a satisfactory experience for participants?

For understanding the participants’ level of satisfaction with the experience, the study explores not only how many used their codes but how many obtained the final certification. Additionally, at the survey, participants were asked about their level of satisfaction with the courses and how useful they perceive the courses they have taken to develop their careers.

As the data in Table 2 remark, the average number of codes requested by a user was 3.84 with a standard deviation (sd) of 2.3 (there was a considerable dispersion, with users requesting from 1 to 25 codes). Most users (38%) asked for five codes, but there were 76 users (4.4%) that asked for more than five. The average number of codes used was 3.2 (sd 2.19). The average number of certificates achieved by a user was 1.8 (sd 1.91), but the courses could be finished several months after the initiative was closed, so the users were still taking courses when the survey was sent. The average number of certificates still in process per user was 1 (sd 1.40).

**Table 2:** Used codes and certificates obtained per number of demanded codes

Demanded codes	% of users	Used codes on avg	Certificates obtained avg	Certificates in process avg
1	16.87%	0.91	0.35	0.22
2	12.76%	1.60	0.75	0.34
3	13.03%	2.41	1.31	0.74
4	8.86%	3.28	1.75	1.05
5	43.45%	4.30	2.34	1.32
6	1.06%	4.94	3.69	1.31
7	0.53%	6.25	2.75	3.50
8	0.40%	6.17	4.33	3.00
9	0.07%	9.00	4.00	5.00
10	2.18%	8.03	4.76	3.00
13	0.07%	13.00	13.00	-
15	0.26%	12.75	4.75	7.00
20	0.33%	16.80	14.20	2.00
22	0.07%	2.00	2.00	-
25	0.07%	22.00	16.00	-
Total	100.00%	3.24	1.76	0.99

Looking at the total data provided by edX, it can be seen that 15,744 of the codes provided to UPV were redeemed and that 5,202 certificates from different institutions were obtained (a 33% completion rate).

In the survey, users affirmed to have obtained an average of 1.8 certificates (sd 1.91), and 84% got at least one certificate (71%) or were in the process to obtain one or more certificates (13%). Around a third of respondents (446 participants, 29%) declared they had finished no MOOC, and more than a half of them (242 participants, 16% of the total respondents) said that they were not in progress of getting one (the other 204 reported that they were still in the process of getting one or more certificates).

1,515 participants answered the survey question about rating their satisfaction with the initiative from zero to five, and the average satisfaction was 4.7/5 with a small dispersion (sd of 0.7). Satisfaction among students was 4.73/5 (sd 0.67) and among workers 4.64/5 (sd 0.73).

The participants were asked to score the quality of each of the MOOCs they had taken, and the average score among respondents was 4.1/5 (sd 1.04). The average given by students is 4.09/5 (sd 1.05), and the average from workers’ answers is 4.14/5 (sd 1.01).

The survey had an open response field for comments, and 296 participants (15.99% of respondents) left their comments. Tagging the answers as positive, negative or neutral in a qualitative coding process, 220 (74.3%) were positive –most thanking for the initiative and asking for its continuation–, 40 (13.5%) were neutral –asking for more time, more codes or making comments about diverse aspects– and 36 (12.1%) negative–some complaining about the deadlines of the courses, others about the quality of a specific course and some about the use of MOOCs to cover for internships-.

The survey also asked users to rate from 0 to 5 how useful they thought that each of the MOOCs they had taken could be for their professional career. The average rating is 3.67/5 with a sd of 1.26. The rating given by students is 3.71/5 (sd 1.24) and by workers 3.59/5 (sd 1.3).

### RQ5: Under what conditions participants would use MOOCs and certifications in the future?

The study asked participants why they joined the initiative to explore the possibility they will use MOOCs in the future for their professional development and under what conditions they would do it. Also, we have asked them if they will use MOOCs in the future, and if they think it is worth paying for MOOC certificates, and what are the reasons that would make them willing to pay for the certificates.

Regarding the reason to join the initiative, participants were asked to score from 0 to 5 some options with the results shown in Table 3.

**Table 3:** Reasons to join the initiative

Reason to join the initiative	average	Students	workers
Improve my resume	3.97	4.04	3.86
Gain new professional skills	4.49	4.49	4.47
Take a course from a prestigious institution	3.26	3.32	3.16
Take advantage of my free time	2.65	2.66	2.65
Learn new things	4.31	4.28	4.35
Curiosity about MOOCs	2.72	2.70	2.74

Differences between students and workers are negligible, with “gaining new professional skills” as the main interest and “learning new things” as the second. The workers are more interested in learning new things and the students in improving their resumes.

98% of all respondents say they will take a MOOC in the future. For the students, the figure is 98.2%, and for the workers, 97.5%

71% of all respondents say they think it is worth paying for the verified certificate of a MOOC. For the students, the figure is 71.4%, and for the workers, 70.7%.

Participants were asked to score from 0 to 5 on these options as the reason to make a MOOC, and the results can be seen in Table 4:

**Table 4:** Reasons to make a MOOC

Reason to make a MOOC	average	Students	workers
The institution that created the course	3.55	3.54	3.57
The Subject	4.12	4.14	4.10
Course Length	2.77	2.78	2.75
Course being part of a professional certificate	3.52	3.55	3.48

**Fig. 6:**
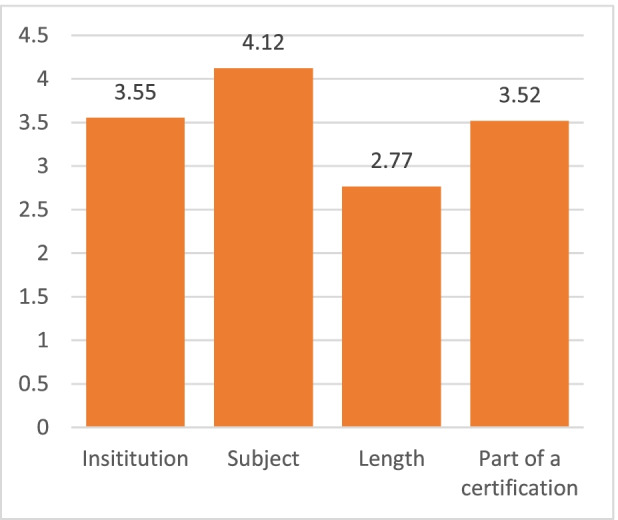
Reasons to pay for a MOOC

Differences between students and workers are negligible, with “Subject” as the main reason to pay for a course and the institution that created the MOOC as the second, as seen in Fig. 6.

## Discussion

This paper presents an analysis of the UPV community experience using the RAP edX initiative during the 2020 lockdown. The experience (free access certificates for most MOOCs edX catalogue) and the 7,712 participants, around 23.4% of the total university community, make this study case an example of the use of MOOCs in a technological HE institution in the western world that is worth being studied.

The analysis leads to the conclusions presented and discussed below.

The distribution of the different groups in the emails of the code requesters and the answers to the survey reflect that employee participation was higher than its quota in the university (they accounted for 18.4% of the requests when they represent around 11.5%, following the current university composition).

Considering the demographic data gathered in the survey, the initiative was participated by a population that reflects the composition of the UPV university community, at least in terms of gender and age. The distribution of gender of the respondents (60% male and 40% female) corresponds almost perfectly with the 38,5% of female students in UPV (Generalitat Valenciana, 2021) and the 40% of women in the UPV workforce (U.P.V., 2021a, b). The distribution of age is also coherent with the three populations (students, employees and alumni) of the universities from the same region (Generalitat Valenciana, 2021).

More employees and alumni answered the survey than students in percentage (14.2% of respondents were alumni when they represented 4.2% of the code requests, and 24% of respondents were employees when they were 18.4% of the code requesters). In addition, the data of the level of education achieved show 41% of degree holders, which is coherent with the 38.2% of answers from employees and alumni.

The significant conclusion about participation shows participation in this local initiative, with these pandemic conditions, has well-reflected the age distribution of universities but has been more frequent among educated people (University staff and alumni), which is similar to participation in MOOCs in other general initiatives. For instance, in the first four years of MOOCs at Harvard and MIT (2012 to 2016), the median age was 29 years, and the percentage of bachelor’s degree holders was 73% (Chuang & Ho, 2016). By its part, in the Macro MOOC learning analytics of global and regional providers (Ruipérez-Valiente et al., 2020), the most common age range across MOOC platforms is 25–35 years, and the percentage of men and people with a bachelor or higher degree of education is even higher for most of the MOOC platforms than the ones we found. Finally, in the Guo and Reinecke (2014) study –4 edX MOOCs with 140,546 students–, they found the mean age was 28, and the most common highest educational level for students was a bachelor’s degree (38%).

Therefore, even if this was a local initiative focussed on a particular population, the MOOCs revalidate its character of resource for educated people.

It is interesting as well to remark that data from the UPV experience regarding gender reflects the composition of its local community; nevertheless, the gender differences are similar to most general MOOC experiences, as the one reflected by Chuang and Ho (2016), where the percentage of female was 33%, or the one of Guo and Reinecke (2014), where most students were men (between 86 and 56% for all four courses). So, the gender gap, studied for technical degrees all over the world (and reflected in the gender composition of the UPV community) is similar to the gender gap in access to MOOCs, as has been studied in Jiang et al. (2018).

The UPV community chose mainly courses from its university (28%) and some of the most prestigious institutions in the world (Harvard, MIT, IBM, Microsoft), followed by courses in Spanish by Spanish-speaking institutions. Most of the favourite courses are focused on Excel and programming languages, like Python and R to work with data, and programming languages, tools very in demand in the business world. These courses are similar to the most popular courses mentioned in the 2021 Coursera Impact report (Yu et al., 2021). Excel and computer programming appear in the first positions of trending skills in several categories.

Data reveal most of the UPV community members had no knowledge or experience with MOOCs. Considering that several emails informing about the initiative were sent to all university community members and that around 23% of the community members participated in the initiative, data concludes the experience increased the awareness of MOOC potential in the community.

This ignorance about MOOCs is similar to what previous studies had reported. In a survey sent to human resource staff of 103 North Carolina (USA) organisations by RTI and Duke University, only 31% answered that they had heard of MOOCs before the survey (Radford et al., 2015). In a survey in Georgia (the country) in 2014, 61% of the students had never heard about MOOCs or taken one of them (Muzafarova & Kaya, 2015). A 2016 study from Nepal reports that 78% of students had never heard about MOOCs (Shakya et al., 2016).

This reality points out one of the main problems of MOOCs: they are unknown by the general public. If these courses were so unknown among the UPV community –the biggest Spanish speaker provider of MOOCs on edX–, the situation in other institutions with less participation in MOOCs and outside HE could be worse.

A 33% completion rate in the UPV experience exceeds over 4 times the 7% average completion rate reported by Khalil and Ebner (2014), or the 7,7% average completion rate for HarvardX and MITx courses reported by Chuang and Ho (2016) and is almost 7 times the 4.65% completion rate of the course studied by Ruipérez-Valiente et al. (2017).

The completion rate is not as high as the 60% completion rate for certificate paying students reported by Chuang and Ho (2016) for edX courses or the 56,7% reported by Goli et al. (2022) for Coursera. Still, it is of the same order of magnitude as the 47% completion rate of certificate track for a MOOC on edX, where free coupons for certificates were offered, and the 41% completion rate for the certificate-paying students of other MOOCs, both reported by Littenberg-Tobias et al. (2020).

According to qualitative (73.6% of positive comments) and quantitative data (average satisfaction with the initiative of 4.7/5 and with course quality of 4.1/5), we can conclude the initiative was perceived as valuable for the University’s community and their satisfaction is similar to the 4.7/5 course rating reported by the biggest MOOC platform, Coursera, in 2021 (Yu et al., 2021). Users were very satisfied with it and rated high the quality of the courses.

UPV community also believes the knowledge they acquire with MOOCs will be valuable for their professional career (3.67/5), which is in line with the 71% of learners from Coursera that report career benefits (Yu et al., 2021).

The vast majority of the UPV community that participated in the survey declared they will take a MOOC in the future, and they think it is worth paying for the verified certificate of a MOOC. The main reason to join the initiative was to gain a new professional skill. The main factors that would make them pay for a MOOC are the course subject and the institution that created the MOOC.

It is noticeable that the main reason to take the courses is the same reported by Breslow et al. (2013) –in their study regarding the first MOOC offered by MIT on edX– and Bayeck (2016) in an exploratory study of MOOC learner’s demographics. But in their study about MOOC use among those who cannot afford formal education, Dillahunt et al. (2014) found the main reason for taking MOOCs was general interest in the topic, with around 80% of the answers of their survey mentioning it. With around 60% of the answers, professional development was “just” in the second position.

## Conclusion

The final reflection of the participation of the UPV community in this initiative is mainly positive. UPV members were very happy with the initiative of providing them free certificates for the MOOCs; they consider the quality of the MOOCs they took as very good and think they will help improve their professional careers. MOOCs are perceived as good tool for professional training.

This study has at least two evident limitations mentioned in the paper. First, it reports the experience of only one institution, and second, even if participation well represents the institution and other similar institutions, the impossibility of a randomised sample in the data collection makes some analysis tricky. Despite these and other limitations, the case study results indicate MOOCs are a highly appreciated tool for professional education in HE, and users value the certificates.

It is important to remark the immense ignorance about the existence of these courses, which would be at the base of some problems cited frequently about MOOCs. They are being used by already educated people (Hansen & Reich , 2015). This situation and the gap in gender in MOOCs access that mirrors the gap in gender in technical universities –at least following the Spanish figures–, remark the importance of doing general campaigns for making broader and more democratic the real access to MOOCs.

Given the success of the UPV courses among the UPV community members, and, as edX gives its members the possibility to offer free codes for their courses to their communities, UPV set up a follow-up initiative to distribute codes UPV courses to UPV members. The university has created a new website using the same system used on the initiative, in which the community members can ask for up to 5 codes for edX courses. When writing this article, one year after setting up the new service, 3,048 codes had been sent to UPV students, staff and faculty, and 774 certificates had been granted (from own records obtained from the edx.org platform and the code distribution app).

UPV has also joined a follow-up initiative of edX called RAP, which offers free certificates of 145 courses to HE institutions around the world until July 2022 (edX, 2020). The mechanism of this new edition is different; edX provides a license to each participant that gives access to the certificates of all included courses. At the moment of writing this article, 2,324 licenses have been distributed by UPV, and participants have obtained 1024 certificates with them (from their own records obtained from the app provided by edX).

Our results emphasise the importance of thinking on MOOCs –as in any initiative regarding the digital transformation of universities or educational institutions– not just as a punctual resource but as a strategical investment (Castañeda, Esteve_Mon & Postigo_Fuentes, In press) that would not only impact the institutional teaching offer but in professionally developing its staff, and on the social compromise of the institutions (Papadimitriou, 2020; Adell Segura, Castañeda Quintero & Esteve Mon, 2018), for critical moments as the COVID-19 crisis, but also beyond them. Governments could finance the certificates of MOOCs to help close the digital gap inside the working institutions and the retraining of the workforce needed (Illanes et al., 2018). Still, there is a need to invest in spreading the word about them to the general public.

Despite the data we present, there is still a lack of educational and institutional research around MOOCs. Higher Education represents one area that has been challenged by the disruption of EdTech (the Educational Technology sector) as a market, and today, it is more important than ever to highlight what we have learnt about scalability in Higher Education from our extensive experience using MOOCs.

In further research, we will address the problems that scalability introduces when dealing with the complex goals of HE and its social justice perspective.

## Data Availability

The data presented in this study are available on request from the corresponding author. The data are not publicly available due to privacy regulations.
